# Randomised immunogenicity trial comparing 2019-2020 recombinant and egg-based influenza vaccines among frequently vaccinated healthcare personnel in Israel

**DOI:** 10.1016/j.ijid.2024.107260

**Published:** 2024-10-10

**Authors:** Ashley L. Fowlkes, Alon Peretz, David Greenberg, Avital Hirsch, Emily T. Martin, Min Z. Levine, Laura Edwards, Sarah Radke, Adam S. Lauring, Jill M. Ferdinands, Chao Zhang, Young M. Yoo, Jacob Dreiher, Gabriella Newes-Adeyi, Eduardo Azziz-Baumgartner, Alicia M. Fry, Arnold S. Monto, Ran Balicer, Mark G. Thompson, Mark A. Katz

**Affiliations:** 1 Influenza Division, Centers for Disease Control and Prevention, Atlanta, Georgia, USA; 2 Rabin Medical Center, Clalit Health Services, Beilinson Campus, Petah Tikva, Israel; 3 Soroka University Medical Center, Clalit Health Services, Be’er Sheva, Israel; 4 Clalit Research Institute, Innovation Division, Clalit Health Services, Tel Aviv, Israel; 5 School of Public Health, University of Michigan, Ann Arbor, Michigan, USA; 6 Abt Associates, Inc., Atlanta, Georgia, USA; 7 National Institute of Health Innovation, University of Auckland, Auckland, New Zealand

**Keywords:** Influenza vaccine, Recombinant, Immunogenicity, Healthcare personnel, Flublok

## Abstract

**Objectives:**

Trivalent inactivated influenza vaccine effectiveness was low in a prospective cohort of healthcare personnel (HCP) in Israel from 2016 to 2019. We conducted a randomised immunogenicity trial of quadrivalent recombinant influenza vaccine (RIV4) and standard-dose inactivated influenza vaccine (IIV4) among frequently and infrequently vaccinated previous cohort participants.

**Methods:**

From October 2019 to January 2020, we enrolled and randomly allocated HCP from two Israeli hospitals to receive IIV4 or RIV4. Hemagglutination inhibition (HAI) antibody titres against 2019-2020 vaccine reference influenza viruses were compared between vaccine groups using geometric mean titre (GMT) ratios from sera collected one-month post-vaccination and by frequency of vaccination in the past 5 years (> 2 vs ≤2).

**Results:**

Among 415 HCP, the GMT ratio comparing RIV4 to IIV4 was 2.0 (95% confidence interval [CI] 1.7-2.7) for A(H1N1)pdm09, 1.6 (95% CI: 1.3-1.9) for A(H3N2), 1.8 (95% CI: 1.4-2.2) for B(Yamagata), and 1.1 (95% CI: 0.9-1.4) for B(Victoria). Similarly, RIV4 elicited higher HAI titres than IIV4 against all 2019-2020 vaccine reference viruses except B(Victoria) among infrequently and frequently vaccinated HCP (lower bound of GMT ratio 95% CIs ≥1.0).

**Conclusion:**

RIV4 had improved immunogenicity for influenza vaccine strains among both infrequent and frequent vaccinees compared to standard-dose IIV4.

**Clinical trials registration:**

NCT04523324

## Background

Annual vaccination with influenza vaccines is either recommended or mandated for healthcare personnel (HCP) in most countries because of their high occupational exposure to viruses and the risk of secondary transmission to patients [1,2]. Influenza vaccines have been shown to reduce the risk of infection among HCP [3]; however, previous studies have found that while frequent influenza vaccination maintains protective antibody titres, frequent vaccination has also been associated with a reduction in serologic response and vaccine effectiveness (VE) [4–6]. Further, egg adaptations during manufacturing of inactivated influenza vaccines can compromise the match of the A(H3N2) vaccine component to circulating viruses [7–9].

Several ‘enhanced’ influenza vaccines have been developed to improve immunogenicity compared to existing vaccines. First, there are enhanced egg-based inactivated vaccines that include adjuvants or use a higher quantity of viral antigen, as in high-dose vaccines [10]. In addition, cell-culture-based vaccines and recombinant vaccines have been developed, which employ technologies that do not require the use of eggs [10]. These vaccines can therefore avoid the issues related to the poor match with A(H3N2) circulating strains observed with egg-based inactivated vaccines. Enhanced influenza vaccines have been shown to be more immunogenic in clinical trials and among repeatedly vaccinated HCP, but few studies have directly compared immunogenicity outcomes among previously vaccinated HCP, and none to date have explored the differential impact among those vaccinated more than three times in the previous five years with those vaccinated less frequently.

Quadrivalent recombinant hemagglutinin-protein influenza vaccine (RIV4; Flublok) is one such enhanced vaccine produced without growth in eggs that is approved for use in several countries, including European Union countries and the United States. Further, the concentration of influenza antigen in RIV4 is higher than standard-dose influenza vaccines (45 *μ*g vs 15 *μ*g of hemagglutinin [HA] per strain, respectively). In clinical trials, RIV4 was demonstrated to be safe, efficacious, and immunogenic [11–13]. Postlicensure evaluations comparing RIV4 to quadrivalent egg-based inactivated influenza vaccines (IIV4) found that RIV4 had an improved immune response against vaccine reference viruses [14–17], findings that supported a recent preferential recommendation in the United States for RIV4, along with other higher dose or adjuvanted vaccines, over standard dose vaccines for adults aged ≥65 years [18]. However, data demonstrating if the same relative advantage in RIV4 immunogenicity is maintained for individuals frequently vaccinated against influenza, such as HCP, are lacking.

In a recent prospective cohort study of HCP in Israel and Peru where mostly egg-based TIV vaccine was used, influenza VE estimates against influenza disease were null during six study seasons [6]. As a follow-up to the studies in Israel and Peru, in order to address whether an enhanced influenza vaccine could provide better protection, we conducted a randomised, open-label immunogenicity trial of RIV4 compared to egg-based, standard dose vaccine (IIV4) among the Israeli HCP participants of the previous cohort study. While vaccine immunogenicity is not equivalent to VE, conducting a relative VE evaluation would have required a very large sample size, for which we did not have the resources. However, previous studies have demonstrated that vaccines with high immunogenicity generate protective levels of antibody and are more likely to be effective [19], and comparative immunogenicity studies are less resource-intensive.

We further evaluated whether the extent of previous influenza vaccination impacted immunogenicity to the two vaccines.

## Methods

### Trial design and participants

This post-licensure randomised controlled immunogenicity trial compared one dose of IIV4 to one dose of RIV4 (ClinicalTrials.gov: NCT04523324). The trial was conducted during the 2019-2020 influenza season at two large hospitals that are part of Clalit Health Services: Rabin Medical Centre, in Petah Tikva, and Soroka Medical Centre, in Be’er Sheva, Israel. We recruited HCP who had previously participated in The Study of Healthcare Personnel with Influenza and other Respiratory Viruses in Israel (SHIRI), a prospective cohort study conducted during the 2016-2017 through 2018-2019 influenza seasons described previously [20]. Briefly, in SHIRI, all HCP employed at the two study hospitals were offered enrolment, regardless of influenza vaccination history or intention to receive influenza vaccine. Approximately 2000 HCP participated in SHIRI when the following influenza vaccines were recommended, but not mandated: trivalent IIV3 during 2016-2017 and 2017-2018, and IIV4 in 2018-2019. During all three influenza seasons, serum was collected from participants at enrolment, 1-month post-vaccination (if vaccinated), and at the end of the influenza season.

At enrolment for the trial, participants completed an electronic survey that included questions about socio-demographic and health characteristics. With the participants’ consent, we extracted additional information, including socio-economic characteristics, comorbidities, and receipt of influenza vaccination during the 5 years prior to the study season, from participants’ electronic medical records (EMR).

Upon availability of the study vaccines, HCP were randomised to receive either IIV4 or RIV4 and asked to return for prevaccination blood collection and vaccine administration. HCP who became pregnant, ill, no longer employed by Clalit Health Services, or no longer wished to continue between enrolment and the time of the vaccination were not vaccinated within the framework of the study and excluded from analysis.

The study protocol was reviewed and approved by the Helsinki committees (institutional review board [IRB]) at both study hospitals and Abt Associates, the coordinating institution. The Centers for Disease Control and Prevention relied on Abt’s IRB. All participants provided written informed consent prior to conducting any study procedures. Study findings are reported in accordance with the Consolidated Standards of Reporting Trials statement guidelines [21].

### Vaccines

Both the IIV4 and the RIV4 vaccines offered to participants contained antigens recommended for the 2019-2020 Northern Hemisphere influenza vaccine strain composition: A/Brisbane/02/2018 (H1N1)pdm09, A/Kansas/14/2017 (H3N2), B/Colorado/06/2017 (Victoria lineage), and B/Phuket/3073/2013 (Yamagata lineage) vaccine antigens. IIV4 was produced from egg-based viral isolates. RIV4 contained recombinant hemagglutinin (HA) proteins based on cell-culture-derived viral isolates.

### Laboratory methods

Participants provided sera pre-vaccination and a targeted 21 to 35 days post-vaccination with a range up to 62 days permitted. Study sera were shipped from Israel to the University of Michigan and U.S. Centers for Disease Control and Prevention (CDC) laboratories on dry ice and stored at −80°C. Sera were tested using HAI assays against the vaccine viruses egg-based A/Brisbane/02/2018 (H1N1)pdm09, A/Kansas/14/2017 (H3N2), B/Colorado/06/2017, and B/Phuket/3073/2013 and cell-based A/Kansas/14/2017 (H3N2) at the University of Michigan [22]. Cell-based A/Kansas/14/2017 (H3N2) virus was propagated in Madin-Darby-Canine kidney (MDCK) SIAT-1 cells. The A(H3N2) antigens were tested for HAI using 0.75% guinea pig erythrocytes with oseltamivir carboxylate. Egg-based viruses were *β*-propiolactone-inactivated for A(H1N1)pdm09 A/Brisbane/02/2018 and ether-inactivated for Victoria B/Colorado/06/2017 and Yamagata B/Phuket/3073/2013. HAI for A(H1N1)pdm09 and B antigens was performed using 0.5% turkey erythrocytes. For a random subset of participants, we also analysed serologic response to both egg- and cell-based A(H3N2) reference viruses using a microneutralisation (MN) assay at the CDC laboratory by incubating serial dilutions of heat inactivated sera with 100 TCID50/well of influenza A(H3N2) viruses. MDCK-SIAT1 cells are then added to the virus-antibody mixture and incubated overnight. The amount of virus in each well was quantified by an enzyme-linked immunosorbent assay (ELISA) and the neutralising antibody titre was defined as the reciprocal of the highest serum dilution that provided ≥50% inhibition of virus infectivity [23].

### Outcomes

The primary outcome measures were post-vaccination HI antibody geometric mean titres (GMTs) against vaccine reference viruses; the seroconversion rate, defined as the proportion of participants that achieved ≥4-fold rises in pre- to post-vaccination antibody titres; mean fold rise (MFR) in pre- to post-vaccination titres; and GMT ratio of post-vaccination titres of RIV4 recipients compared to IIV4 recipients. Titres less than 1:10 were considered undetectable and reported as 1:5 for analysis purposes. We further assessed seroprotection as the percentage of participants with post-vaccination titres ≥1:40 associated with 50% protection against influenza illness [24], or ≥1:160 identified as a more relevant threshold in children and frequently used as a more conservative definition of seroprotection in evaluations among adults [24].

As secondary outcomes, we conducted subgroup analyses comparing post-vaccination serologic responses among HCP who were infrequently vaccinated (0-2 times) during the previous 5 seasons to HCP frequently vaccinated (3-5 times) in the previous 5 seasons. We also compared the serologic response according to receipt of IIV during only the prior 2018-2019 influenza season.

### Data analysis

All statistical analyses of antibody titres were conducted using log base-2 transformed titre data; results were then back-transformed to the original scale for ease of interpretation. We compared seroconversion and seroprotection rates using χ2 tests, and expressed the absolute difference in seroconversion rates between vaccines. We estimated the GMT, MFR in pre- to post-vaccination titres, and GMT ratios of post-vaccination titres of RIV4 recipients compared to IIV4 recipients using separate linear mixed models that accounted for repeated measures of participants and adjusted for age, hospital, and immunocompromised. For subgroup comparisons based on influenza vaccination history, linear mixed models calculated GMT ratios comparing post-vaccination titres within RIV4 and IIV4 vaccine groups and comparing post-vaccination titres of for each subgroup among RIV4 recipients compared to IIV4 recipients. Unadjusted analyses are provided in Supplementary Tables 1–3. Statistical significance was indicated by non-overlapping 95% CIs, or a *p*-value <0.05. All analyses were conducted with SAS software, version 9.4 (SAS Institute), and R software, version 4.0.2 (R Foundation for Statistical Computing).

## Results

### Participant characteristics

**Figure 1.**
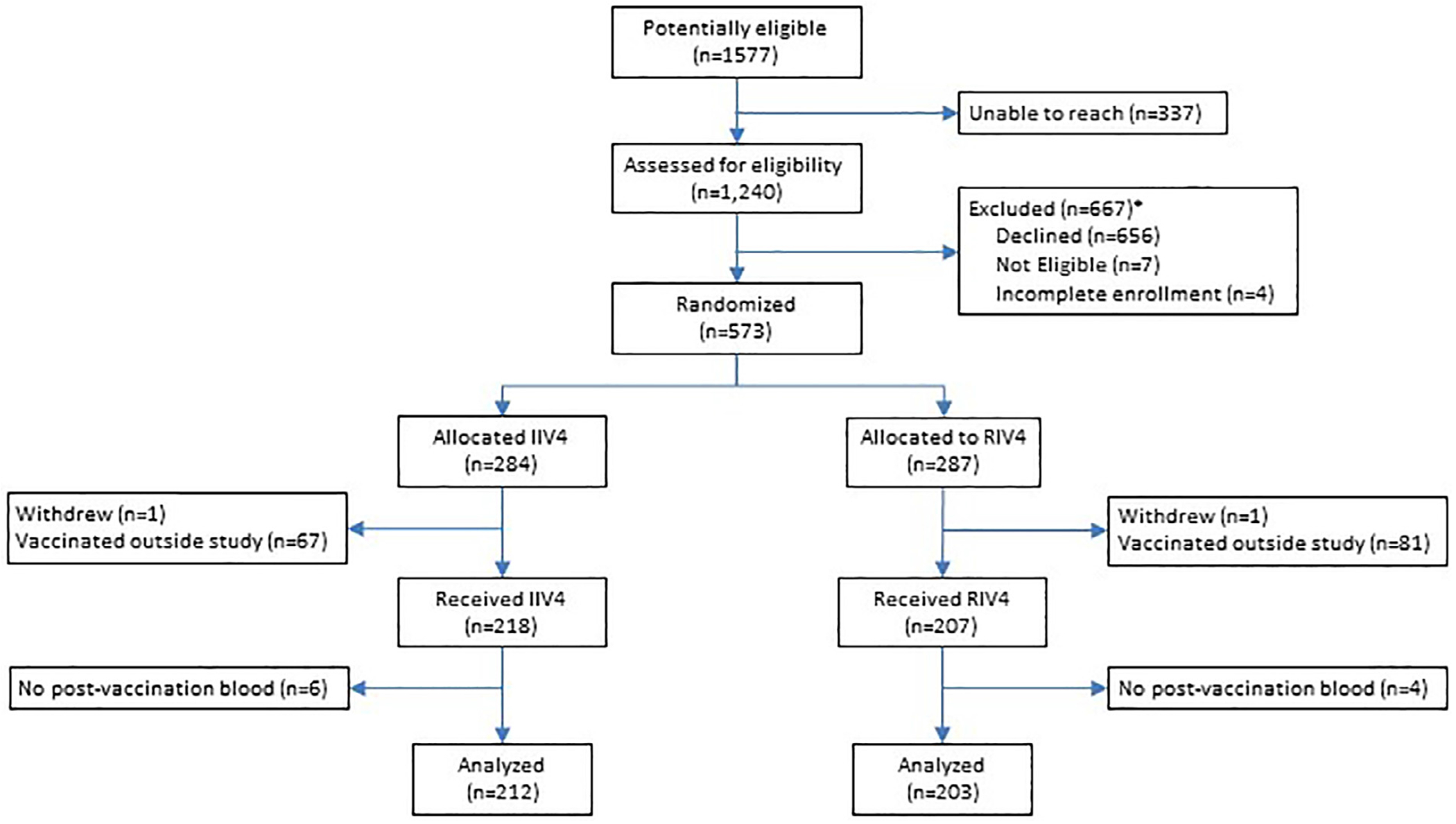
CONSORT diagram of healthcare personnel enrolled in a randomized, controlled immunogenicity trial of quadrivalent recombinant-hemagglutinin and egg-grown influenza vaccines, Israel, 2019-2020. Among 1,577 participants of a previous cohort study, 1,240 healthcare personnel were reached; 656 declined participation, 7 were not eligible due to no longer being employed in the recruiting hospital, not currently working, or pregnant, and 4 consented, but did not complete enrollment.

Of 1240 SHIRI HCP contacted, 573 (46.2%) met inclusion criteria, enrolled in the study, and were randomised to receive IIV4 and RIV4 (Figure 1). We excluded 147 (25.8%) participants vaccinated outside of the study and 2 (0.3%) participants who withdrew between enrolment and vaccine administration; 10 (1.7%) participants without post-vaccination serum were also excluded. The final analytical population included 415 HCP, of whom 212 received IIV4 and 203 received RIV4 (Table 1). RIV4 recipients were slightly older than IIV4 recipients (median age 47 [IQR 38-57] vs 43 [IQR 36-52] years, respectively). Vaccination groups otherwise had similar demographic and health characteristics. Overall, 299 HCP (72%) were frequently vaccinated against influenza prior to the study, and 329 HCP (79%) received influenza vaccine in the 2018-2019 season. Post-vaccination sera were collected at a median of 28 days (IQR 25-32) following vaccination; the range was 21-46 days with only 13 HCP providing blood after day 35. The number of days from vaccination to post-vaccination serum collection did not differ by vaccination group.

### Antibody response at one month post vaccination

Pre-vaccination titres for each of the antigens tested did not differ significantly among IIV4 and RIV4 groups (Table 2). Overall, HCP who received RIV4 had a more robust antibody response against all vaccine reference viruses, compared to recipients of IIV4, as measured by seroconversion rates one-month post-vaccination titres and by mean fold rise.

### Response to A(H1N1)pdm09 vaccine virus

**Figure 2.**
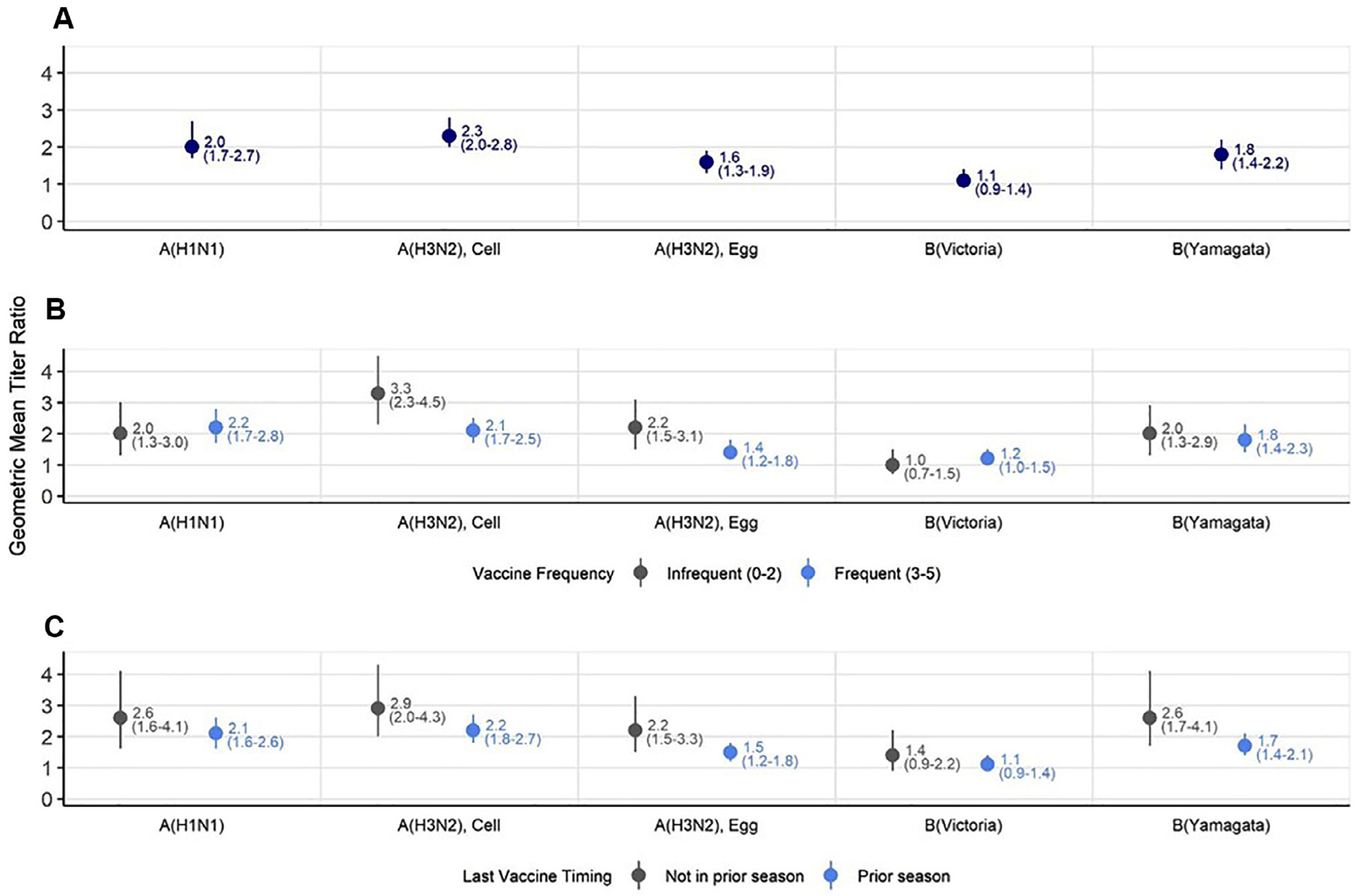
Geometric mean titer ratios comparing humoral antibody responses 1-month post-vaccination with 2019-2020 quadrivalent recombinant and egg-based influenza vaccines among all participants (a), and by frequency of influenza vaccination over the past 5 seasons (b) and vaccination during the prior 2018–2019 season (c). Geometric mean titer ratios were calculated by linear mixed model or generalized estimating equation (GEE for GMT ≥160) after controlling for age, hospital, and immunocompromised status. Frequency of vaccinations were categorized as frequent and infrequent: frequently vaccinated, defined as receiving influenza vaccine in 3–5 of the past 5 seasons, compared with those infrequently vaccinated, defined as receiving influenza vaccine in ≤2 of the past 5 seasons.

Compared to IIV4 recipients, RIV4 recipients achieved a higher seroconversion rate against the A(H1N1)pdm09 vaccine reference virus (52.8 vs 23.8%; absolute difference: 29.0% [95% CI: 20.1-37.8%]), had a higher mean fold rise (4.0 [95% CI: 3.2-5.0] vs 1.9 [95% CI: 1.5-2.3]), and achieved higher post-vaccination titres (GMT ratio: 2.0 [95% CI: 1.7-2.7]) (Table 2, Figure 2A). The vast majority of RIV4 recipients and IIV4 recipients had post-vaccination antibody titres ≥40 against A(H1N1)pdm09 (95.6% vs 87.3%; absolute difference: 8.3% [95% CI: 3.0-13.6%]). In contrast, 66.3% of RIV4 recipients had post-vaccination antibody titres ≥160 against A(H1N1)pdm09 compared to only 38.9% of IIV4 recipients [absolute difference: 27.3% (95% CI: 18.2-36.5%)].

### Response to egg- and cell-based A(H3N2) vaccine viruses

Similarly, RIV4 recipients compared to IIV4 recipients had higher seroconversion rates to both egg-based A(H3N2) (57.2% vs 34.8%; absolute difference: 22.4% [95% CI: 13.1-31.8%]) and cell-based A(H3N2) (77.4% vs 41.0%; absolute difference: 36.9% [95% CI: 28.3-45.6%]). The post-vaccination GMT ratio comparing RIV4 to IIV4 against egg-based A(H3N2) virus was 1.6 (95% CI: 1.3-1.9); against cell-based virus the GMT ratio was 2.3 (95% CI: 2.0-2.8). Both IIV4 and RIV4 recipients had higher pre- and post-vaccination titres to egg-based A(H3N2) compared to cell-based virus (Table 2, Figure 2A). Among RIV4 recipients, 98% had post-vaccination titres against egg-based A(H3N2) virus ≥40 and IIV4 recipients had 97% (absolute difference: 0.37% [95% CI: −2.7-3.5%]). Against cell-based A(H3N2) virus, 96% of RIV4 participants and 88% IIV4 recipients had post-vaccination titres ≥40 (absolute difference: 7.9% [95% CI: 2.8-13.0%]). Compared to IIV4 recipients, a higher percentage of RIV4 recipients had GMT ≥160 against egg-based A(H3N2) [absolute difference: 16.7% (95% CI: 8.5-25.0%)] and against the cell-based virus (absolute difference: 34.9% [95% CI: 25.8-43.9%]).

We conducted microneutralisation assays against egg- and cell-based A(H3N2) for a random subset of post-vaccination sera from participants who received IIV4 (n=29) or RIV4 (n=27) (Supplementary Table 4). The post-vaccination GMT ratio induced by RIV4 compared to IIV4 was 2.2 (95% CI=1.2-3.7) and 2.1 (95% CI=1.2-3.9) against egg- and cell-gown A(H3N2), respectively.

### Response to B(Yamagata) and B(Victoria) vaccine viruses

Compared to participants who received IIV4, RIV4 participants had a higher seroconversion rate against the B(Yamagata) vaccine virus (absolute difference: 22.6% [95% CI: 14.4-30.8]), a 1.8-fold (95% CI: 1.4-2.2) higher post-vaccination GMT, and a higher percentage of RIV4 participants than IIV4 recipients achieved very high post-vaccination titres (≥160) (absolute difference: 17.2% [95% CI: 8.0-26.3]) (Table 2, Figure 2). Similarly, RIV4 recipients had a higher seroconversion rate against B(Victoria) compared with IIV4 recipients (18.0% vs 9.0%; absolute difference: 8.9 [95% CI: 2.3-15.5]). The post-vaccination GMT ratio was slightly higher (1.1-fold [95% CI: 0.9-1.4]), but differences in other endpoints were not statistically significant (Table 2, Figure 2A).

### Antibody response by frequency of vaccination over 5 prior seasons

Within vaccine groups, there was a significant mean fold rise in titres from pre- to post-vaccination, but post-vaccination GMTs among frequently vaccinated HCP were generally lower than infrequently vaccinated HCPs (Supplementary Table 5). Among frequently vaccinated IIV4 recipients compared with those infrequently vaccinated, post-vaccination GMTs were significantly lower against A(H1N1)pdm09, B(Victoria); among RIV4 recipients, post-vaccination GMTs were significantly lower against all vaccine viruses except B(Victoria). Comparing RIV4 post-vaccination antibody responses to IIV4 antibody responses, post-vaccination GMTs were significantly higher for RIV4 in both subgroups against all vaccine reference viruses (GMT ratios range, 1.4-fold to 3.3-fold higher) with the exception of B(Victoria) (GMT ratios 1.0 (95% CI: 0.07-1.5) (Table 3, Figure 2B). Against B(Victoria), post-vaccination titres were slightly higher among frequently vaccinated HCP (GMT ratio: 1.2 [95% CI: 1.0-1.5]) and did not differ infrequently vaccinated HCP.

### Antibody response by prior season vaccination status

In our stratified analysis of HCP vaccinated with IIV3 during the prior 2018-2019 season and those unvaccinated during the prior influenza season, we found a similar trend; the pre- to post-vaccination mean fold rises were statistically significant, but post-vaccination GMTs were generally lower among HCP vaccinated in the prior season than those unvaccinated (Supplementary Table 6). Post-vaccination titres against influenza A viruses and B(Yamagata) were significantly higher for RIV4 recipients than for IIV4 recipients, regardless of prior season vaccination (Table 4). The GMT ratio of RIV4 to IIV4 post-vaccination titres ranged from 1.5 (95% CI: 1.2-1.8) against egg-based A(H3N2) in HCP with prior season vaccination to 2.9 (95% CI: 2.0-4.3) against cell-based A(H3N2) for participants without prior-season vaccination (Figure 2C). Similar to findings during the 5 previous seasons, differences in antibody responses did not reach statistical significance against B(Victoria), regardless of prior season vaccination.

## Discussion

In this randomised immunogenicity trial among HCP, most of whom had a history of frequent influenza vaccination, RIV4 elicited more robust anti-HA antibody titres compared to standard-dose IIV4 against influenza A(H1N1)pdm09, A(H3N2), and B(Yamagata) vaccine reference viruses one-month after vaccination. Vaccination with RIV4 substantially increased seroconversion by 22 to 37% percentage points compared to IIV4 for A(H1N1)pdm09 and A(H3N2) viruses. The point estimates of vaccine HA immunogenicity measures against influenza B(Victoria) were higher for RIV4 compared to IIV4, though the increases were not significant. Furthermore, while both IIV4 and RIV4 vaccines induced a significant rise in mean antibody titres regardless of prior influenza vaccination history, recipients of RIV4 compared to IIV4 had higher post-vaccination titres among HCP frequently vaccinated over the past five seasons and those vaccinated in the season prior (2018-2019). These findings in a frequently vaccinated population with high occupational exposure to influenza virus add to mounting evidence from post-licensure observational studies that RIV offers superior immunogenicity compared to IIV among adults [14,15,25,26].

Reduced immune response to egg-based vaccines has emerged as a major concern about IIV4, and concerns about reduced protection from IIV4 have been found to contribute to reduced vaccine uptake [27,28]. In our study, both RIV4 and IIV4 elicited a significant immune response against the egg-based A/Kansas/14/2017 (H3N2) that was 1.6-fold higher for RIV4 recipients compared to IIV4. Previous studies have suggested that increased RIV4 immunogenicity may result from the higher antigen dose in the vaccine, a broader vaccine-induced antibody response to the purified recombinant HA, or improved affinity of antibodies elicited by recombinant HA to viral HA [25,29]. We were further able to assess immunogenicity against cell-based A(H3N2) virus, which is more likely to be similar to circulating viruses than the egg-based vaccine virus and has been previously used as an endpoint in other RIV4 immunogenicity trials [30,31]. Measured against the cell-based A(H3N2) virus, post vaccination titres for RIV were a further 2.3-fold higher than for IIV4. While the amount of antigen in RIV4 was originally determined to be comparable to the amount of antigen in the standard egg-derived HA [32], the magnitude of our observed effects is similar to those in a recent comparison of RIV4 and IIV4 among HCP in the United States [16] and similar to the improved immunogenicity observed in studies of other enhanced vaccines, such as high-dose and adjuvanted influenza vaccines [15]. We further found that RIV4 immunogenicity was higher than IIV4 for influenza B components; this finding differs from recent trials that have found RIV4 had a similar immune response to B virus vaccine components as IIV4 [16,33].

In this study, we had a unique opportunity to compare immune response to IIV4 or RIV4 by vaccination history, in contrast to other influenza immunogenicity studies of HCP, which were either limited to only frequently vaccinated adults [16] or included participants where vaccine history was not examined [14]. We found that, importantly, the MFR in titres post-vaccination was significant in all groups, indicating that all groups mounted a significant response to both influenza vaccines. However, RIV4 elicited significantly higher immunogenicity than IIV4 among HCP irrespective of whether they were vaccinated with any IIV the previous season or how frequently they were vaccinated during the previous 5 years. While our study still demonstrated that frequently vaccinated HCP had lower titres to RIV4 than those infrequently vaccinated, our findings contribute to the growing evidence suggesting that enhanced influenza vaccines like RIV4 may be able to elicit a sufficiently high antibody response to overcome the effects of blunting associated with frequent vaccination [4,5,15,17,34]. These findings are supported by a recent study trial demonstrating that the higher post-vaccination HI antibody titre induced by RIV4 was sustained over two consecutive seasons [26], and consistent with results from clinical trials of RIV [30,31,33].

This study has several limitations. First, due to a shipping delay in study vaccines, several HCP were vaccinated outside of the study between enrolment and vaccination. Although the samples size was reduced, both study vaccine groups were impacted equally. Second, despite randomisation and regardless of exclusions, RIV4 recipients were significantly older than IIV4 recipients. As the median ages only varied by 4 years and the participating HCP were largely younger than age 65, the possible impacts of immunosenescence were likely minimised and a higher immune response among RIV4 recipients compared with IIV4 was still able to be demonstrated. Third, microneutralisation assays against egg- and cell-based A(H3N2) were conducted for only a random subset of participants, which lowered the precision of effect. Nonetheless, the pattern of effects using microneutralisation was similar to those observed with HI. Fourth, while the study protocol indicated the optimal timing for post-vaccination serum collection was within 21 to 35 days, a range up to 62 days was permitted to allow for operational challenges such as participant leave and holidays. As a result, 13 HCP provided blood between days 36 to 46, which did not vary by vaccination group. Fifth, we were not able to assess immune responses against the influenza neuraminidase (NA) glycoprotein as RIV4 does not include NA and IIV4 includes varying amounts of NA that are not quantified in manufacturing.

We found that RIV4 produced higher immunogenicity than IIV4 across a multitude of immunogenicity measures. While vaccine immunogenicity is not equivalent to vaccine efficacy or effectiveness, a recent study demonstrated higher effectiveness of RIV4 in preventing influenza as compared to IIV4 among persons 50 to 60 years old [32]. Further studies evaluating effectiveness for additional populations and outcomes are needed to inform vaccine policy recommendations and determine the sustainability of immunogenicity upon repeated RIV4 vaccination in subsequent seasons. Nonetheless, the superior immunogenicity of RIV4 among HCP suggests it may be an effective option in efforts to optimise the protective benefits of influenza vaccines among HCP.

## Supplementary Material

Supplement

## Figures and Tables

**Table 1:** Characteristics of trial participants by vaccine group, Israel, 2019-2020.

Characteristic	IIV4 n = 212		RIV4 n = 203		*p*-value ^a^
	N	%	N	%	
Age					
18-44 years	119	56.1	85	41.9	**0.004**
≥45 years	93	43.9	118	58.1	
Sex					
Male	71	33.5	70	34.5	0.831
Female	141	66.5	133	65.5	
Ethnicity					
Jewish, not Ultra-Orthodox	194	91.5	181	89.2	0.560
Jewish, Ultra-Orthodox	3	1.4	6	3.0	
Arab	15	7.1	16	7.9	
Socioeconomic status^b^					
Low	26	12.3	18	8.9	0.450
Middle	67	31.6	72	35.5	
High	119	56.1	113	55.7	
Hospital					
Soroka Medical Centre	102	48.1	84	41.4	0.168
Rabin Medical Centre	110	51.9	119	58.6	
Urbanicity ^c^					
Urban	191	90.5	186	92.5	0.463
Rural	20	9.5	15	7.5	
Occupation					
Physician	65	30.7	50	24.6	0.368
Nurse, medical therapist, technician	106	50.0	113	55.7	
Medical assistant and support staff	41	19.3	40	19.7	
Works ≥30 hours per week					
No	8	3.8	11	5.4	0.423
Yes	204	95.8	192	94.6	
Supplemental insurance					
No	9	4.2	7	3.4	0.673
Yes	203	95.8	196	96.6	
≥1 chronic medical condition					
No	162	76.4	146	71.9	0.295
Yes	50	23.6	57	28.1	
Immunocompromised					
No	212	100.0	200	98.5	0.116
Yes	0	0.0	3	1.5	
HIV infection					
No	212	100.0	201	99.0	0.239
Yes	0	0.0	2	1.0	
Body Mass Index Category ^c^					
Underweight (<18.5)	2	1.0	4	2.0	0.759
Normal (18.5 to <25)	84	40.4	85	42.9	
Overweight (25 to <30)	77	37.0	67	33.8	
Obese (≥30)	45	21.6	42	21.2	
Self-reported health					
Excellent	61	28.8	66	32.5	0.195
Very Good	102	48.1	80	39.4	
Good/Fair/Poor	49	23.1	57	28.1	
Smoking history ^c^					
Never smoked	143	68.1	154	77.4	0.068
Past smoker	42	20.0	24	12.1	
Current smoker	25	11.9	21	10.6	
Influenza vaccine received in prior season (2018-2019)					
No evidence of vaccination	8	3.8	6	3.0	0.645
Vaccinated	204	96.2	197	97.0	
Influenza vaccines received in previous 5 seasons					
0-2	61	28.8	55	27.1	0.703
3-5	151	71.2	148	72.9	
Influenza during any year of SHIRI					
No	183	86.3	177	87.2	0.794
Yes	29	13.7	26	12.8	
Influenza vaccinated during any year of SHIRI					
No	13	6.1	12	5.9	0.925
Yes	199	93.9	191	94.1	

Abbreviations: IIV4, Quadrivalent inactivated influenza vaccine; RIV4, Quadrivalent recombinant influenza vaccine; SHIRI, Study of Healthcare Personnel with Influenza and other Respiratory Viruses in Israel.

a *P*-values were calculated using non-parametric Fisher’s exact test when the expected value was <5. Statistically significant findings are shown in bold.

b Socioeconomic characteristics, current and past medical conditions, medical care utilisation and vaccination history was extracted from the Clalit Health Services database.

c Urbanicity was not reported for 2 (0.6%) IIV4 recipients and 2 (1.0%) RIV4 recipients. Body mass index category was not available for 10 (3.0%) IIV4 (recipients and 5 (2.5%) RIV4 recipients. 4 (1.2%) IIV4 participants and 4 (2.0%) RIV4 participants did not respond to smoking history.

**Table 2:** Humoral immune responses pre- and post-vaccination among recipients of quadrivalent inactivated egg-based influenza vaccine (IIV4) and quadrivalent recombinant influenza vaccine (RIV4) measured by hemagglutination inhibition assay against influenza vaccine reference viruses ^a^, Israel, 2019-2020.

Hemagglutination inhibition assay outcome measures^b^	IIV4 n = 212				RIV4 n = 203				RIV4 vs IIV4 Post-Vaccination				*P*-value
	Pre-vaccination		Post-vaccination		Pre-vaccination		Post-vaccination		Difference (95% CI)		GMT ratio (95% CI)		
**Influenza A(H1N1)pdm09**													
GMT (95% CI)	44.1	(38.1-51.1)	82.9	(71.6-96.1)	44.5	(38.2-51.7)	178.4	(153.5-207.5)			**2.0**	**(1.7-2.7)**	<**0.001**
Mean-Fold Rise (95% CI)			**1.9**	**(1.5-2.3)**			**4.0**	**(3.2-5.0)**					
Seroconversion, % (95% CI)			23.8	(18.2-29.5)			52.8	(46.1-59.5)	**29.0**	**(20.1-37.8)**			
GMT ≥40, % (95% CI)	68.4	(62.1-74.7)	87.3	(82.8-91.4)	67.0	(60.5-73.5)	95.6	(92.7-98.4)	**8.3**	**(3.0-13.6)**			
GMT ≥160, % (95% CI)	20.8	(14.9-25.5)	38.9	(32.4-45.4)	20.6	(15.1-26.0)	66.3	(59.8-72.7)	**27.3**	**(18.2-36.5)**			
**Influenza A(H3N2), Egg-based**													
GMT (95% CI)	62.4	(54.7-71.2)	152.8	(133.9-174.3)	60.3	(52.7-69.0)	246.9	(215.8-282.5)			**1.6**	**(1.3-1.9)**	<**0.001**
Mean-Fold Rise (95% CI)			**2.4**	**(2.0-2.9)**			**4.1**	**(3.4-5.0)**					
Seroconversion, % (95% CI)			34.8	(28.4-41.3)			57.2	(50.5-64.0)	**22.4**	**(13.1-31.8)**			
GMT ≥40, % (95% CI)	84.0	(79.0-88.9)	97.2	(94.9-99.4)	75.9	(70.0-81.8)	97.5	(95.4-99.7)	0.37	(−2.7-3.5)			
GMT ≥160, % (95% CI)	26.2	(20.4-32.0)	63.4	(57.1-69.7)	29.2	(23.1-35.3)	80.2	(74.8-85.5)	**16.7**	**(8.5-25.0)**			
**Influenza A(H3N2), Cell-based**													
GMT (95% CI)	30.7	(27.2-34.6)	80.8	(71.6-91.1)	31.1	(27.5-35.2)	189.2	(167.3-214.0)			**2.3**	**(2.0-2.8)**	<**0.001**
Mean-Fold Rise (95% CI)			**2.6**	**(2.2-3.1)**			**6.1**	**(5.1-7.2)**					
Seroconversion, % (95% CI)			41.0	(33.9-47.1)			77.4	(71.8-83.0)	**36.9**	**(28.3-45.6)**			
GMT ≥40 (95% CI)	56.1	(49.5-62.8)	88.2	(83.9-92.6)	58.6	(51.9-65.4)	96.1	(93.4-98.7)	**7.9**	**(2.8-13.0)**			
GMT ≥160 (95% CI)	2.5	(0.4-4.6)	30.6	(24.4-36.8)	3.7	(1.1-6.3)	65.8	(59.3-72.3)	**34.9**	**(25.8-43.9)**			
**Influenza B(Victoria)**													
GMT (95% CI)	54.0	(46.7-62.3)	78.3	(67.9-90.4)	48.5	(41.9-56.2)	90.3	(78.0-104.6)			1.1	(0.9-1.4)	0.175
Mean-Fold Rise (95% CI)			**1.5**	**(1.2-1.8)**			**1.9**	**(1.5-2.3)**					
Seroconversion, % (95% CI)			9.0	(5.3-13.0)			18.0	(12.7-23.3)	**8.9**	**(2.3-15.5)**			
GMT ≥40, % (95% CI)	70.7	(64.5-76.8)	83.0	(77.8-89.7)	67.1	(60.6-73.6)	82.9	(79.7-89.7)	1.8	(−5.4-9.0)			
GMT ≥160, % (95% CI)	24.2	(18.5-29.9)	34.0	(27.4-40.1)	19.9	(14.4-25.4)	40.1	(33.4-46.9)	6.4	(−2.9-15.7)			
**Influenza B(Yamagata)**													
GMT (95% CI)	68.7	(59.7-79.0)	105.7	(91.9-121.7)	71.7	(62.1-82.7)	196.2	(170.0-226.5)			**1.8**	**(1.4-2.2)**	<**0.001**
Mean-Fold Rise (95% CI)			**1.5**	**(1.3-1.9)**			**2.7**	**(2.2-3.4)**					
Seroconversion, % (95% CI)			14	(9.3-18.7)			36.6	(30.0-43.3)	**22.6**	**(14.4-30.8)**			
GMT ≥40, % (95% CI)	83.0	(78.0-88.1)	90.1	(86.1-94.1)	83.7	(78.7-88.8)	97.0	(94.7-99.4)	**7.0**	**(2.3-11.6)**			
GMT ≥160, % (95% CI)	31.6	(25.4-37.8)	50.0	(43.2-56.7)	30.1	(24.0-36.2)	67.1	(60.9-73.4)	**17.2**	**(8.0-26.3)**			

Abbreviations: IIV4, Quadrivalent inactivated influenza vaccine; RIV4, Quadrivalent recombinant influenza vaccine; GMT, Geometric mean titre;

a Influenza vaccine reference viruses: A/Brisbane/02/2018 (H1N1)pdm09, A/Kansas/14/2017 (H3N2), B/Colorado/06/2017 (Victoria lineage), and B/Phuket/3073/2013 (Yamagata lineage) vaccine antigens.

b GMT, Mean-Fold Rise in GMT, and GMT ratios were calculated using linear mixed models adjusting for age, hospital, and immunocompromised status. Statistically significant differences are shown in bold.

**Table 3:** Antibody responses pre- and post-vaccination with quadrivalent recombinant protein influenza vaccine (RIV4) and quadrivalent inactivated egg-based influenza vaccine (IIV4) against influenza vaccine reference viruses^a^ among participants who received influenza vaccine infrequently (0-2 seasons) or frequently (3-5 seasons) during the prior five seasons^b^.

Frequency of vaccinations in prior five influenza seasons ^b , c^	IIV4		RIV4		Post-vaccination GMT RIV4 vs IIV4 (95% CI)	*P*-value
	N	Post-vaccination GMT (95% CI)	N	Post-vaccination GMT (95% CI)		
**Influenza A(H1N1)pdm09**						
Frequent (3-5)	151	71.8 (60-85)	148	159.4 (134-190)	**2.2 (1.7 - 2.8)**	<**0.001**
Infrequent (0-2)	61	120.1 (91-158)	55	238.8 (179-318)	**2.0 (1.3 - 3.0)**	<**0.001**
**Influenza A(H3N2), Egg-based**						
Frequent (3-5)	151	149.5 (128-175)	148	216.6 (185-253)	**1.4 (1.2 - 1.8)**	**0.001**
Infrequent (0-2)	61	161.9 (127-207)	55	350.0 (271-453)	**2.2 (1.5 - 3.1)**	<**0.001**
**Influenza A(H3N2), Cell-based**						
Frequent (3-5)	151	80.1 (70-92)	148	166 (144-192)	**2.1 (1.7 - 2.5)**	<**0.001**
Infrequent (0-2)	61	82.6 (66-103)	55	269 (212-340)	**3.3 (2.3 - 4.5)**	<**0.001**
**Influenza B (Victoria)**						
Frequent (3-5)	151	67.8 (57-80)	148	82.6 (69-98)	**1.2 (1.0 - 1.5)**	0.108
Infrequent (0-2)	61	113.1 (87-148)	55	113.5 (86-150)	1.0 (0.7 - 1.5)	0.985
**Influenza B (Yamagata)**						
Frequent (3-5)	151	91.9 (78-108)	148	167.4 (142-198)	**1.8 (1.4 - 2.3)**	<**0.001**
Infrequent (0-2)	61	151.3 (117-196)	55	296.6 (226-389)	**2.0 (1.3 - 2.9)**	<**0.001**

Abbreviations: Quadrivalent inactivated influenza vaccine (IIV4), Quadrivalent recombinant influenza vaccine (RIV4), Geometric mean titre (GMT).

a Influenza vaccine reference viruses: A/Brisbane/02/2018 (H1N1)pdm09, A/Kansas/14/2017 (H3N2), B/Colorado/06/2017 (Victoria lineage), and B/Phuket/3073/2013 (Yamagata lineage) vaccine antigens.

b Frequency of vaccinations were categorised as frequent and infrequent: frequently vaccinated, defined as receiving influenza vaccine in 3-5 of the past five seasons, compared with those infrequently vaccinated, defined as receiving influenza vaccine in ≤2 of the past five seasons.

c GMT and GMT ratios were calculated by linear mixed models adjusting for age, hospital, and immunocompromised status. Statistically significant findings are shown in bold.

**Table 4:** Antibody responses pre- and post-vaccination with quadrivalent recombinant protein influenza vaccine (RIV4) and quadrivalent inactivated egg-based influenza vaccine (IIV4) against influenza vaccine reference viruses^a^ according to influenza vaccine receipt during the previous season.

Prior influenza season (2018-2019) vaccination status^b^	IIV4		RIV4		Post-vaccination GMT RIV4 vs IIV4 (95% CI)	*P*-value
	N	Post-vaccination GMT (95% CI)	N	Post-vaccination GMT (95% CI)		
**Influenza A(H1N1)pdm09**						
Prior season	166	80.2 (68-95)	163	165 (140-196)	**2.1 (1.6 - 2.6)**	<**0.001**
Not in prior season	46	93.9 (68-129)	40	243 (174-341)	**2.6 (1.6 - 4.1)**	<**0.001**
**Influenza A(H3N2), Egg-based**						
Prior season	166	152 (131-176)	163	228 (196-264)	**1.5 (1.2 - 1.8)**	<**0.001**
Not in prior season	46	156 (118-207)	40	344 (254-465)	**2.2 (1.5 - 3.3)**	<**0.001**
**Influenza A(H3N2), Cell-based**						
Prior season	166	81.6 (71-93)	163	181 (158-208)	**2.2 (1.8 - 2.7)**	<**0.001**
Not in prior season	46	78.0 (60-101)	40	227 (172-299)	**2.9 (2.0 - 4.3)**	<**0.001**
**Influenza B (Victoria)**						
Prior season	166	73.3 (62-86)	163	81.1 (69-95)	1.1 (0.9 - 1.4)	0.391
Not in prior season	46	99.8 (73-136)	40	139 (100-194)	1.4 (0.9 - 2.2)	0.145
**Influenza B (Yamagata)**						
Prior season	166	101 (86-118)	163	172 (147-202)	**1.7 (1.4 - 2.1)**	<**0.001**
Not in prior season	46	126 (94-171)	40	332 (241-458)	**2.6 (1.7 - 4.1)**	<**0.001**

Abbreviations: IIV4, Quadrivalent inactivated influenza vaccine; RIV4, Quadrivalent recombinant influenza vaccine; GMT, Geometric mean titre.

a Influenza vaccine reference viruses: A/Brisbane/02/2018 (H1N1)pdm09, A/Kansas/14/2017 (H3N2), B/Colorado/06/2017 (Victoria lineage), and B/Phuket/3073/2013 (Yamagata lineage) vaccine antigens.

b GMT and GMT ratios were calculated using linear mixed models adjusting for age, hospital, and immunocompromised status. Statistically significant findings are shown in bold.
