# Supplementary material for: Randomised immunogenicity trial comparing 2019-2020 recombinant and egg-based influenza vaccines among frequently vaccinated healthcare personnel in Israel
Source: Int J Infect Dis. Author manuscript; Available in PMC 2025 Mar 6. (PMC11883642; doi:10.1016/j.ijid.2024.107260)
Supplement: Supplement [file NIHMS2057069-supplement-Supplement.docx]

**Unadjusted Analyses**

**Supplementary Table 1.** Humoral immune responses pre- and post-vaccination among recipients of quadrivalent inactivated egg-based influenza vaccine (IIV4) and quadrivalent recombinant influenza vaccine (RIV4) measured by hemagglutination inhibition assay against influenza vaccine reference viruses, Israel, 2019-2020

| **Hemagglutination Inhibition Assay Outcome Measures** | **IIV4** | | | | | | |  | | **RIV4** | | | | | | | |  | | **RIV4 vs. IIV4 Post-Vaccination** | | | | | | | | |
| --- | --- | --- | --- | --- | --- | --- | --- | --- | --- | --- | --- | --- | --- | --- | --- | --- | --- | --- | --- | --- | --- | --- | --- | --- | --- | --- | --- | --- |
|  | **n = 212** | | | | | | |  | | **n = 203** | | | | | | | |  | |  |  |  |  |  |  |  |  |  |
|  | Pre-Vaccination | | | Post-Vaccination | | | |  | | Pre-Vaccination | | | | | Post-Vaccination | | | |  | | Difference (95% CI) | | | | GMT Ratio (95% CI) | | | |
| **Influenza A(H1N1)pdm09** |  |  | |  | |  | |  | |  | |  | |  | |  | |  | |  | |  | |  | |  | |  |
| GMT (95% CI) | 45.4 | (39.1 – 52.8) | | 85.4 | | (73.5 – 99.3) | |  | | 43.1 | | (37.0 – 50.2) | | 173.1 | | (148.5 – 201.7) | |  | |  | |  | | **2.0** | | **(1.6 – 2.5)** | |  |
| Mean-Fold Rise (95% CI) | REF |  | | 1.9 | | (1.5 – 2.3) | |  | |  | |  | | 4.0 | | (3.2 – 5.0) | |  | |  | |  | |  | |  | |  |
| Seroconversion, % (95% CI) |  |  | | 22.2 | | (16.6 – 27.8) | |  | |  | |  | | 53.7 | | (46.8 – 60.6) | |  | | **31.5** | | **(22.7 – 40.4)** | |  | |  | |  |
| GMT ≥40, % (95% CI) | 68.4 | (62.1 – 74.7) | | 87.3 | | (82.8 – 91.4) | |  | | 67.0 | | (60.5 – 73.5) | | 95.6 | | (92.7 – 98.4) | |  | | **8.3** | | **(3.0 – 13.6)** | |  | |  | |  |
| GMT ≥160, % (95% CI) | 20.8 | (15.3 – 26.2) | | 39.6 | | (33.0 – 46.2) | |  | | 19.7 | | (14.2 – 25.2) | | 65.5 | | (59.0 – 72.1) | |  | | **25.9** | | **(16.6 – 35.2)** | |  | |  | |  |
|  |  |  | |  | |  | |  | |  | |  | |  | |  | |  | |  | |  | |  | |  | |  |
| **Influenza A(H3N2), Egg-Grown** | | |  | |  | |  | |  | |  | |  | |  | |  | |  | |  | |  | |  | |  |  |
| GMT (95% CI) | 63.6 | (55.7 – 72.7) | | 155.9 | | (136.4 – 178.0) | |  | | 59 | | (51.5 – 67.6) | | 241.9 | | (211.1 – 277.0) | |  | |  | |  | | **1.6** | | **(1.3 – 1.9)** | |  |
| Mean-Fold Rise (95% CI) |  |  | | 2.4 | | (2.0 – 3.0) | |  | |  | |  | | 4.1 | | (3.4 – 5.0) | |  | |  | |  | |  | |  | |  |
| Seroconversion, % (95% CI) |  |  | | 35.4 | | (28.9 – 42.2) | |  | |  | |  | | 56.7 | | (49.8 – 63.5) | |  | | **21.3** | | **(11.9 – 30.7)** | |  | |  | |  |
| GMT ≥40, % (95% CI) | 84.0 | (79.0 – 88.9) | | 97.2 | | (94.9 – 99.4) | |  | | 75.9 | | (70.0 – 81.8) | | 97.5 | | (95.4 – 99.7) | |  | | 0.37 | | (-2.7 – 3.5) | |  | |  | |  |
| GMT ≥160, % (95% CI) | 26.4 | (20.5 – 32.4) | | 63.7 | | (57.2 – 70.2) | |  | | 28.1 | | (21.9 – 34.3) | | 79.3 | | (73.7 – 84.9) | |  | | **15.6** | | **(7.1 – 24.2)** | |  | |  | |  |
|  |  |  | |  | |  | |  | |  | |  | |  | |  | |  | |  | |  | |  | |  | |  |
| **Influenza A(H3N2), Cell-Grown** | | |  | |  | |  | |  | |  | |  | |  | |  | |  | |  | |  | |  | |  |  |
| GMT (95% CI) | 31.3 | (27.7 – 35.4) | | 82.4 | | (72.9 – 93.1) | |  | | 30.4 | | (26.8 – 34.5) | | 185.3 | | (163.4 – 210.1) | |  | |  | |  | | **2.2** | | **(1.0 – 2.7)** | |  |
| Mean-Fold Rise (95% CI) |  |  | | 2.6 | | (2.6 – 3.1) | |  | |  | |  | | 6.1 | | (5.1 – 7.3) | |  | |  | |  | |  | |  | |  |
| Seroconversion, % (95% CI) |  |  | | 41.0 | | (34.4 – 47.7) | |  | |  | |  | | 76.4 | | (70.5 – 82.2) | |  | | **35.3** | | **(26.5 – 44.2)** | |  | |  | |  |
| GMT ≥40 (95% CI) | 56.1 | (49.5 – 62.8) | | 88.2 | | (83.9 – 92.6) | |  | | 58.6 | | (51.9 – 65.4) | | 96.1 | | (93.4 – 98.7) | |  | | **7.9** | | **(2.8 – 13.0)** | |  | |  | |  |
| GMT ≥160 (95% CI) | 2.4 | (0.3 – 4.4) | | 30.7 | | (24.5 – 36.9) | |  | | 3.5 | | (0.9 – 6.0) | | 65.5 | | (59.0 – 72.1) | |  | | **34.9** | | **(25.8 – 43.9)** | |  | |  | |  |
|  |  |  | |  | |  | |  | |  | |  | |  | |  | |  | |  | |  | |  | |  | |  |
| **Influenza B(Victoria)** |  |  | |  | |  | |  | |  | |  | |  | |  | |  | |  | |  | |  | |  | |  |
| GMT (95% CI) | 54.4 | (47.1 – 62.8) | | 79 | | (68.4 – 91.3) | |  | | 48.1 | | (41.7 - 55.4) | | 89.5 | | (77.7 - 103.2) | |  | |  | |  | | 1.1 | | (0.9 – 1.4) | |  |
| Mean-Fold Rise (95% CI) |  |  | | 1.5 | | (1.2 – 1.8) | |  | |  | |  | | 1.9 | | (1.5 – 2.3) | |  | |  | |  | |  | |  | |  |
| Seroconversion, % (95% CI) |  |  | | 9.0 | | (5.1 – 12.8) | |  | |  | |  | | 18.2 | | (12.9 – 23.5) | |  | | **9.3** | | **(2.7 – 15.8)** | |  | |  | |  |
| GMT ≥40, % (95% CI) | 70.8 | (64.6 – 76.9) | | 83 | | (78.0 – 88.1) | |  | | 67 | | (60.5 – 73.5 ) | | 84.7 | | (79.8 – 89.7) | |  | | 1.7 | | (-5.4 – 8.8) | |  | |  | |  |
| GMT ≥160, % (95% CI) | 24.5 | (18.7 – 30.3) | | 34 | | (27.6 – 40.3) | |  | | 19.7 | | (14.2 – 25.2) | | 39.9 | | (33.2 – 46.6) | |  | | 5.9 | | (-3.3 – 15.2) | |  | |  | |  |
|  |  |  | |  | |  | |  | |  | |  | |  | |  | |  | |  | |  | |  | |  | |  |
| **Influenza B(Yamagata)** |  |  | |  | |  | |  | |  | |  | |  | |  | |  | |  | |  | |  | |  | |  |
| GMT (95% CI) | 70.4 | (61.1 – 81.2) | | 108.4 | | (94.0 – 125.1) | |  | | 69.8 | | (60.3 – 80.6) | | 191.1 | | (165.2 – 221.0) | |  | |  | |  | | **1.8** | | **(1.4 – 2.2)** | |  |
| Mean-Fold Rise (95% CI) |  |  | | 1.5 | | (1.3 – 1.9) | |  | |  | |  | | 2.7 | | (2.2 – 3.4) | |  | |  | |  | |  | |  | |  |
| Seroconversion, % (95% CI) |  |  | | 13.7 | | (9.1 – 18.3) | |  | |  | |  | | 37 | | (30.3 – 43.6) | |  | | **23.3** | | **(15.2 – 31.4)** | |  | |  | |  |
| GMT ≥40, % (95% CI) | 83.0 | (78.0 – 88.1) | | 90.1 | | (86.1 – 94.1) | |  | | 83.7 | | (78.7 – 88.8) | | 97.0 | | (94.7 – 99.4) | |  | | **7.0** | | **(2.3 – 11.6)** | |  | |  | |  |
| GMT ≥160, % (95% CI) | 32.6 | (26.2 – 38.9) | | 50.9 | | (44.2 – 57.7) | |  | | 28.6 | | (22.4 – 34.8) | | 65.5 | | (59.0 – 72.1) | |  | | **14.6** | | **(5.2 – 24.0)** | |  | |  | |  |

Abbreviations: Quadrivalent inactivated influenza vaccine (IIV4), Quadrivalent recombinant influenza vaccine (RIV4), Geometric mean titer (GMT).

**Supplemental Table 2.** Antibody responses pre- and post-vaccination with quadrivalent recombinant protein influenza vaccine (RIV4) and quadrivalent inactivated egg-based influenza vaccine (IIV4) against influenza vaccine reference viruses among participants who received influenza vaccine infrequently (0-2 seasons) or frequently (3-5 seasons) during the prior five seasons

|  | **IIV4** | | | | |  | **RIV4** | | | | | **RIV4 vs. IIV4** |
| --- | --- | --- | --- | --- | --- | --- | --- | --- | --- | --- | --- | --- |
| **Frequency of vaccinations in prior 5 influenza seasons*†** | **N** | **Pre-vaccination GMT (95% CI)** | **Post-vaccination GMT (95% CI)** | **Mean Fold Rise  (95% CI)** | **Post-vaccination GMT ratio  (95% CI)** |  | **N** | **Pre-vaccination GMT (95% CI)** | **Post-vaccination GMT (95% CI)** | **Mean Fold Rise  (95% CI)** | **Post-vaccination GMT ratio  (95% CI)** | **Post-vaccination GMT (95% CI)** |
| **Influenza A/Brisbane/02/2018 (H1N1)pdm09** | | | | | |  |  |  |  |  |  |  |
| Frequent (3-5) | 151 | 40.0 (34-48) | 71.7 (60-85) | 1.8 (1.4-2.3) | **0.5 (0.4-0.8)** |  | 148 | 43.3 (36-52) | 152.7 (128-182) | 3.5 (2.7-4.5) | **0.6 (0.4-0.9)** | 2.1 (1.7-2.7) |
| Infrequent (0-2) | 61 | 62.3 (47-82) | 131.9 (100-174) | 2.1 (1.4-3.1) | REF |  | 55 | 42.6 (32-57) | 242.5 (182-324) | 5.7 (3.8-8.6) | REF | 1.8 (1.2-2.7) |
|  |  |  |  |  |  |  |  |  |  |  |  |  |
| **Influenza A/Kansas/14/2017 (H3N2), Egg** | | | | | |  |  |  |  |  |  |  |
| Frequent (3-5) | 151 | 62.7 (54-73) | 149.4 (128-174) | 2.4 (1.9-2.9) | 0.9 (0.7-1.1) |  | 148 | 57.6 (49-68) | 209.9 (179-246) | 3.6 (2.9-4.6) | **0.6 (0.4-0.8)** | 1.4 (1.1-1.8) |
| Infrequent (0-2) | 61 | 65.9 (52-83) | 173.2 (135-221) | 2.6 (1.9-3.7) | REF |  | 55 | 63.0 (48-83) | 353.9 (273-459) | 5.6 (3.9-8.1) | REF | 2.0 (1.4-2.9) |
|  |  |  |  |  |  |  |  |  |  |  |  |  |
| **Influenza A/Kansas/14/2017 (H3N2), Cell** | | | | | |  |  |  |  |  |  |  |
| Frequent (3-5) | 151 | 31.4 (27-36) | 80.0 (70-92) | 2.6 (2.1-3.1) | 0.9 (0.7-1.2) |  | 148 | 29.9 (26-35) | 161 (139-186) | 5.4 (4.4-6.6) | **0.6 (0.4-0.8)** | 2.0 (1.6-2.5) |
| Infrequent (0-2) | 61 | 31.2 (25-39) | 88.6 (71-111) | 2.8 (2.1-3.9) | REF |  | 55 | 31.9 (25-41) | 272 (214-345) | 8.5 (6.0-11.9) | REF | 3.1 (2.2-4.2) |
|  |  |  |  |  |  |  |  |  |  |  |  |  |
| **Influenza B/Colorado/06/2017 (Victoria)** | | | | | |  |  |  |  |  |  |  |
| Frequent (3-5) | 151 | 49.2 (42-58) | 67.8 (57-80) | 1.4 (1.1-1.7) | **0.6 (0.4-0.8)** |  | 148 | 48.4 (41-57) | 81.9 (69-97) | 1.7 (1.3-2.2) | 0.7 (0.5-1.0) | 1.2 (1.0-1.5) |
| Infrequent (0-2) | 61 | 69.8 (54-91) | 115.1 (89-150) | 1.6 (1.1-2.4) | REF |  | 55 | 47.7 (36-63) | 113.9 (86-150) | 2.4 (1.6-3.5) | REF | 1.0 (0.7-1.5) |
|  |  |  |  |  |  |  |  |  |  |  |  |  |
| **B/Phuket/3073/2013 (Yamagata)** | | | | | |  |  |  |  |  |  |  |
| Frequent (3-5) | 151 | 63.0 (53-74) | 91.8 (78-108) | 1.5 (1.2-1.8) | **0.6 (0.4-0.8)** |  | 148 | 70.2 (59-83) | 161.5 (137-191) | 2.3 (1.8-2.9) | **0.5 (0.4-0.7)** | 1.8 (1.4-2.2) |
| Infrequent (0-2) | 61 | 92.7 (71-120) | 163.7 (126-213) | 1.8 (1.2-2.5) | REF |  | 55 | 68.8 (52-91) | 300.5 (228-396) | 4.4 (2.9-6.4) | REF | 1.8 (1.3-2.7) |

Abbreviations: Quadrivalent inactivated influenza vaccine (IIV4), Quadrivalent recombinant influenza vaccine (RIV4), Geometric mean titer (GMT).

* Frequency of vaccinations were categorized as frequent and infrequent: frequently vaccinated, defined as receiving influenza vaccine 3 – 5 of the past 5 seasons, compared with those infrequently vaccinated, defined as receiving influenza vaccine ≤2 of the past 5 season

**Supplemental Table 3.** Antibody responses pre- and post-vaccination with quadrivalent recombinant protein influenza vaccine (RIV4) and quadrivalent inactivated egg-based influenza vaccine (IIV4) against influenza vaccine reference viruses according to influenza vaccine receipt during the previous season

|  | **IIV4** | | | | | |  | **RIV4** | | | | | | **RIV4 vs. IIV4** |
| --- | --- | --- | --- | --- | --- | --- | --- | --- | --- | --- | --- | --- | --- | --- |
| **Prior Influenza Season (2018-2019) Vaccination Status** | **N** | **Pre-vaccination**  **GMT (95% CI)** | **Post-vaccination GMT (95% CI)** | **Mean Fold Rise**  **(95% CI)** | **Post-vaccination GMT Ratio**  **(95% CI)*** |  | | **N** | **Pre-vaccination GMT (95% CI)** | **Post-vaccination GMT (95% CI)** | **Mean Fold Rise (95% CI)** | **Post-vaccination GMT Ratio**  **(95% CI)*** | **Post-vaccination GMT (95% CI)** | |
| **Influenza A/Brisbane/02/2018 (H1N1)pdm09** | | | | | |  | |  |  |  |  |  |  | |
| Prior season | 166 | 45.9 (39-54) | 81.3 (68-96) | 1.8 (1.4-2.3) | 0.8 (0.6-1.1) |  | | 163 | 44.9 (38-53) | 159 (134-189) | 3.6 (2.8-4.5) | 0.7 (0.5-1.0) | 2.0 (1.5-2.5) | |
| Not in prior season | 46 | 43.8 (32-61) | 101.8 (74-140) | 2.3 (1.5-3.7) | REF |  | | 40 | 36.7 (26-52) | 243 (172-343) | 6.6 (4.1-10.8) | REF | 2.4 (1.5-3.8) | |
|  |  |  |  |  |  |  | |  |  |  |  |  |  | |
| **Influenza A/Kansas/14/2017 (H3N2), Egg** | | | | |  |  | |  |  |  |  |  |  | |
| Prior season | 166 | 61.8 (53-72) | 153 (132-178) | 2.5 (2.0-3.0) | 0.9 (0.7-1.3) |  | | 163 | 58 (50-67) | 221 (191-258) | 3.8 (3.1-4.7) | 0.6 (0.5-0.9) | 1.4 (1.2-1.8) | |
| Not in prior season | 46 | 70.9 (53-94) | 165 (124-219) | 2.3 (1.6-3.5) | REF |  | | 40 | 63.9 (46-88) | 343 (253-466) | 5.4 (3.5-8.3) | REF | 2.1 (1.4-3.1) | |
|  |  |  |  |  |  |  | |  |  |  |  |  |  | |
| **Influenza A/Kansas/14/2017 (H3N2), Cell** | | | | |  |  | |  |  |  |  |  |  | |
| Prior season | 166 | 31.3 (27-36) | 82.4 (72-95) | 2.6 (2.2-3.2) | 1.0 (0.7,1.3) |  | | 163 | 29.8 (26-34) | 176 (153-203) | 5.9 (4.8-7.2) | 0.8 (0.6-1.1) | 2.1 (1.7-2.6) | |
| Not in prior season | 46 | 31.4 (24-41) | 82.4 (63-107) | 2.6 (1.8-3.8) | REF |  | | 40 | 33.1 (25-44) | 226 (171-300) | 6.8 (4.6-10.2) | REF | 2.7 (1.9-4.0) | |
|  |  |  |  |  |  |  | |  |  |  |  |  |  | |
| **Influenza B/Colorado/06/2017 (Victoria)** | | | | |  |  | |  |  |  |  |  |  | |
| Prior season | 166 | 54.3 (46-64) | 73.6 (63-86) | 1.4 (1.1-1.7) | 0.7 (0.5-1.0) |  | | 163 | 47.0 (40-55) | 80.3 (68-95) | 1.7 (1.3-2.2) | 0.6 (0.4-0.8) | 1.1 (0.9-1.4) | |
| Not in prior season | 46 | 54.9 (41-74) | 101.8 (75-138) | 1.9(1.2-2.8) | REF |  | | 40 | 52.8 (38-74) | 139 (100-193) | 2.6 (1.6-4.2) | REF | 1.4 (0.8-2.1) | |
|  |  |  |  |  |  |  | |  |  |  |  |  |  | |
| **B/Phuket/3073/2013 (Yamagata)** | | | | |  |  | |  |  |  |  |  |  | |
| Prior season | 166 | 73.0 (63-86) | 102 (87-120) | 1.4 (1.1-1.7) | 0.8 (0.5-1.1) |  | | 163 | 69.2 (59-81) | 167 (142-196) | 2.4 (1.9-3.0) | 0.5 (0.4-0.7) | 1.6 (1.3-2.1) | |
| Not in prior season | 46 | 61.9 (46-84) | 136 (100-184) | 2.2 (1.4-3.4) | REF |  | | 40 | 72.1 (52-100) | 331 (239-459) | 4.6 (2.9-7.3) | REF | 2.4 (1.6-3.8) | |

Abbreviations: Quadrivalent inactivated influenza vaccine (IIV4), Quadrivalent recombinant influenza vaccine (RIV4), Geometric mean titer (GMT).

**Microneutralization Assay Results**

**Supplemental Table 4.** Humoral immune responses measured by microneutralization assay against egg-grown and cell-grown influenza A(H3N2) vaccine reference viruses among recipients of a quadrivalent inactivated egg-based influenza vaccine and a quadrivalent recombinant protein influenza vaccine.

|  | IIV4 | | | |  | RIV4 | | | |  | **RIV4 vs. IIV4 Post-Vaccination** | | | |
| --- | --- | --- | --- | --- | --- | --- | --- | --- | --- | --- | --- | --- | --- | --- |
|  | **n = 29** | | | |  | **n = 27** | | | |  | Absolute Difference (95% CI) | | GMT Ratio (95% CI) | |
| **Microneutralization Assay Outcome Measures** | Pre-Vaccination | | Post-Vaccination | |  | Pre-Vaccination | | Post-Vaccination | |  |  |  |  |  |
| **Influenza A(H3N2) Egg-Grown** |  |  |  |  |  |  |  |  |  |  |  |  |  |  |
| GMT (95% CI) | 74.5 | (50-110) | 160.0 | (108-237) |  | 68.6 | (46-103) | 223.4 | (149-336) |  |  |  | 2.1 | (1.2-3.7) |
| Mean-Fold Rise in GMT (95% CI) |  |  | 2.1 | (1-4) |  |  |  | 3.3 | (2-6) |  |  |  |  |  |
| Seroconversion, % (95% CI) |  |  | 31.0 | (14-48) |  |  |  | 51.9 | (33-71) |  | 20.8 | (-4.5-46.1) |  |  |
| GMT ≥40 (95% CI) | 86.2 | (74-99) | 93.1 | (84-100) |  | 74.1 | (58-91) | 92.6 | (83-100) |  | -0.5 | (-14.0-13.0) |  |  |
| GMT ≥160 (95% CI) | 31.0 | (14-48) | 69.0 | (52-86) |  | 33.3 | (16-51) | 74.1 | (58-91) |  | 5.1 | (-18.5-28.7) |  |  |
|  |  |  |  |  |  |  |  |  |  |  |  |  |  |  |
| **Influenza A(H3N2) Cell-Grown** |  |  |  |  |  |  |  |  |  |  |  |  |  |  |
| GMT (95% CI) | 31.5 | (22-46) | 90.2 | (62-132) |  | 30.9 | (21-6) | 191.5 | (129-284) |  |  |  | 2.2 | (1.2-3.9) |
| Mean-Fold Rise in GMT (95% CI) |  |  | 2.9 | (2-5) |  |  |  | 6.2 | (4-11) |  |  |  |  |  |
| Seroconversion, % (95% CI) |  |  | 41.4 | (24-59) |  |  |  | 74.1 | (58-91) |  | 32.7 | (8.3-57.1) |  |  |
| GMT ≥40 (95% CI) | 55.2 | (37-73) | 93.1 | (84-100) |  | 51.8 | (33-71) | 92.6 | (83-100) |  | -0.5 | (-14.0-13.0) |  |  |
| GMT ≥160 (95% CI) | 3.5 | (0-10) | 37.9 | (20-56) |  | 7.4 | (0-17) | 63.0 | (45-81) |  | 25.0 | (-0.3-50.4) |  |  |

Abbreviations: Quadrivalent inactivated influenza vaccine (IIV4), Quadrivalent recombinant influenza vaccine (RIV4), Geometric mean titer (GMT).

**Adjusted Analysis Within-Vaccine Group**

**Supplemental Table 5.** Antibody responses pre- and post-vaccination with quadrivalent recombinant protein influenza vaccine (RIV4) and quadrivalent inactivated egg-based influenza vaccine (IIV4) against influenza vaccine reference viruses among participants who received influenza vaccine infrequently (0-2 seasons) or frequently (3-5 seasons) during the prior five seasons.

|  | **IIV4** | | | | |  | **RIV4** | | | | |
| --- | --- | --- | --- | --- | --- | --- | --- | --- | --- | --- | --- |
| **Frequency of vaccinations in prior 5 influenza seasons*†** | **N** | **Pre-vaccination** | **Post-vaccination** | **Pre- to Post-vaccination** | **Frequent vs. infrequent post-vaccination** |  | **N** | **Pre-vaccination** | **Post-vaccination** | **Pre- to Post-vaccination** | **Frequent vs. infrequent post-vaccination** |
|  |  | **GMT (95% CI)** | **GMT (95% CI)** | **MFR (95% CI)** | **GMT Ratio (95% CI)** |  |  | **GMT (95% CI)** | **GMT (95% CI)** | **MFR (95% CI)** | **GMT ratio (95% CI)** |
| **Influenza A(H1N1)pdm09** | | | | | |  |  |  |  |  |  |
| Frequent (3-5) | 151 | 40.1 (34-48) | 71.8 (60-85) | **1.8 (1.4-2.3)** | **0.6 (0.4-0.8)** |  | 148 | 45.2 (38-54) | 159.4 (134-190) | **3.5 (2.7-4.5)** | **0.5(0.5-0.9)** |
| Infrequent (0-2) | 61 | 56.7 (43-75) | 120.1 (91-158) | **2.1 (1.4-3.1)** | REF |  | 55 | 42.0 (32-56) | 238.8 (179-318) | **5.7 (3.8-8.5)** | REF |
|  |  |  |  |  |  |  |  |  |  |  |  |
| **Influenza A(H3N2), Egg-based** | | | | | |  |  |  |  |  |  |
| Frequent (3-5) | 151 | 62.8 (54-73) | 149.5 (128-175) | **2.4 (1.9-2.9)** | 0.9 (0.7-1.2) |  | 148 | 59.4 (51-69) | 216.6 (185-253) | **3.6 (2.9-4.5)** | **0.6 (0.5-0.8)** |
| Infrequent (0-2) | 61 | 61.6 (48-79) | 161.9 (127-207) | **2.6 (1.9-3.7)** | REF |  | 55 | 62.2 (48-81) | 350.0 (271-453) | **5.6 (3.9-8.1)** | REF |
|  |  |  |  |  |  |  |  |  |  |  |  |
| **Influenza A(H3N2), Cell-based** | | | | | |  |  |  |  |  |  |
| Frequent (3-5) | 151 | 31.4 (27-36) | 80.1 (70-92) | **2.6 (2.1-3.1)** | 1.0 (0.7-1.3) |  | 148 | 30.9 (27-36) | 166 (144-192) | **5.4 (4.4-6.6)** | **0.6 (0.5-0.8)** |
| Infrequent (0-2) | 61 | 29.1 (23-36) | 82.6 (66-103) | **2.8 (2.1-3.9)** | REF |  | 55 | 31.5 (25-40) | 269 (212-340) | **8.5 (6.1-11.9)** | REF |
|  |  |  |  |  |  |  |  |  |  |  |  |
| **Influenza B (Victoria)** | | | | | |  |  |  |  |  |  |
| Frequent (3-5) | 151 | 49.2 (42-58) | 67.8 (57-80) | **1.4 (1.1-1.7)** | **0.6 (0.4-0.8)** |  | 148 | 48.6 (41-58) | 82.6 (69-98) | **1.7 (1.3-2.2)** | 0.7 (0.5-1.0) |
| Infrequent (0-2) | 61 | 68.6 (53-90) | 113.1 (87-148) | **1.7 (1.1-2.4)** | REF |  | 55 | 47.6 (36-63) | 113.5 (86-150) | **2.4 (1.6-3.5)** | REF |
|  |  |  |  |  |  |  |  |  |  |  |  |
| **Influenza B (Yamagata)** | | | | | |  |  |  |  |  |  |
| Frequent (3-5) | 151 | 63.1 (54-74) | 91.9 (78-108) | **1.5 (1.2-1.8)** | **0.6 (0.4-0.8)** |  | 148 | 72.7 (62-86) | 167.4 (142-198) | **2.3 (1.8-2.9)** | **0.6 (0.4-0.8)** |
| Infrequent (0-2) | 61 | 85.7 (66-111) | 151.3 (117-196) | **1.8 (1.2-2.5)** | REF |  | 55 | 67.9 (52-89) | 296.6 (226-389) | **4.4 (3.0-6.4)** | REF |
| Abbreviations: Quadrivalent inactivated influenza vaccine (IIV4), Quadrivalent recombinant influenza vaccine (RIV4), Geometric mean titer (GMT). | | | | | | | | | |  |  |
| * Frequency of vaccinations were categorized as frequent and infrequent: frequently vaccinated, defined as receiving influenza vaccine 3 – 5 of the past 5 seasons, compared with those infrequently vaccinated, defined as receiving influenza vaccine ≤2 of the past 5 season | | | | | | | | | | | |

**Supplemental Table 6.** Antibody responses pre- and post-vaccination with quadrivalent recombinant protein influenza vaccine (RIV4) and quadrivalent inactivated egg-based influenza vaccine (IIV4) against influenza vaccine reference viruses according to influenza vaccine receipt during the previous season.

|  | **IIV4** | | | | |  | **RIV4** | | | | |  |
| --- | --- | --- | --- | --- | --- | --- | --- | --- | --- | --- | --- | --- |
| **Prior Influenza Season (2018-2019) Vaccination Status** | **N** | **Pre-vaccination** | **Post-vaccination** | **Pre- to Post-vaccination MFR** | **Frequent vs. infrequent post-vaccination** |  | **N** | **Pre-vaccination** | **Post-vaccination** | **Pre- to Post-vaccination** | **Frequent vs. infrequent post-vaccination** | |
|  |  | **GMT (95% CI)** | **GMT (95% CI)** | **MFR (95% CI)** | **GMT Ratio (95% CI)** |  |  | **GMT (95% CI)** | **GMT (95% CI)** | **MFR (95% CI)** | **GMT ratio (95% CI)** | |
| **Influenza A(H1N1)pdm09** |  |  |  |  |  |  |  |  |  |  |  | |
| Prior season | 166 | 45.2 (38-53) | 80.2 (68-95) | 1.8 (1.4-2.2) | 0.9 (0.6-1.2) |  | 163 | 46.6 (39-55) | 165 (140-196) | 3.6 (2.8-4.5) | 0.7 (0.5-1.0) | |
| Not in prior season | 46 | 40.4 (29-55) | 93.9 (68-129) | 2.3 (1.5-3.6) | REF |  | 40 | 36.8 (26-52) | 243 (174-341) | 6.6 (4.1-10.7) | REF | |
|  |  |  |  |  |  |  |  |  |  |  |  | |
| **Influenza A(H3N2), Egg-based** |  |  |  |  |  |  |  |  |  |  |  | |
| Prior season | 166 | 61.2 (53-71) | 152 (131-176) | 2.5 (2.0-3.1) | 1.0 (0.7-1.3) |  | 163 | 59.3 (51-69) | 228 (196-264) | 3.8 (3.1-4.7) | 0.7 (0.5-0.9) | |
| Not in prior season | 46 | 67.2 (51-89) | 156 (118-207) | 2.3 (1.6-3.5) | REF |  | 40 | 64.0 (47-87) | 344 (254-465) | 5.4 (3.4-8.5) | REF | |
|  |  |  |  |  |  |  |  |  |  |  |  | |
| **Influenza A(H3N2), Cell-based** |  |  |  |  |  |  |  |  |  |  |  | |
| Prior season | 166 | 31.0 (27-35) | 81.6 (71-93) | 2.6 (2.2-3.2) | 1.0 (0.8,1.4) |  | 163 | 30.5 (27-35) | 181 (158-208) | 5.9 (4.9-7.2) | 0.8 (0.6-1.1) | |
| Not in prior season | 46 | 29.7 (23-39) | 78.0 (60-101) | 2.6 (1.8-3.8) | REF |  | 40 | 33.1 (25-44) | 227 (172-299) | 6.8 (4.6-10.1) | REF | |
|  |  |  |  |  |  |  |  |  |  |  |  | |
| **Influenza B (Victoria)** |  |  |  |  |  |  |  |  |  |  |  | |
| Prior season | 166 | 54.1 (46-64) | 73.3 (62-86) | 1.4 (1.1-1.7) | 0.7 (0.5-1.0) |  | 163 | 47.4 (40-56) | 81.1 (69-95) | 1.7 (1.4-2.2) | 0.6 (0.4-0.8) | |
| Not in prior season | 46 | 53.8 (40-73) | 99.8 (73-136) | 1.9(1.2-2.8) | REF |  | 40 | 52.8 (38-73) | 139 (100-194) | 2.6 (1.7-4.2) | REF | |
|  |  |  |  |  |  |  |  |  |  |  |  | |
| **Influenza B (Yamagata)** |  |  |  |  |  |  |  |  |  |  |  | |
| Prior season | 166 | 72.1 (62-84) | 101 (86-118) | 1.4 (1.1-1.7) | 0.8 (0.6-1.1) |  | 163 | 71.4 (61-84) | 172 (147-202) | 2.4 (1.9-3.0) | 0.5 (0.4-0.7) | |
| Not in prior season | 46 | 57.8 (43-78) | 126 (94-171) | 2.2 (1.4-3.3) | REF |  | 40 | 72.3 (52-100) | 332 (241-458) | 4.6 (2.9-7.2) | REF | |
| Abbreviations: Quadrivalent inactivated influenza vaccine (IIV4), Quadrivalent recombinant influenza vaccine (RIV4), Geometric mean titer (GMT). | | | | | | | | |  |  |  | |
